# 

**Published:** 1896-06

**Authors:** 


CONTENTS—JUNE, 1896.—VOL. XI., NO. 12.

ORIGINAL COMMUNICATIONS—

Notes on Some of the Newer Methods of Treatment of Nervous and Mental Disease. Frederick Peterson, M. D., New York . . . . . 659

Some Cases of Osteomyelitis. W. R. Blailock, M. D., McGregor, Texas . . . . . . . . . . . . . . . . . . . . . . . 675

Some of the Mistakes of Surgical Gynecology. T. A. Stoddard, M. D., Pueblo, Col . . . . . . . . . . . . . . . . . . . . 680

SOCIETY NOTES—

North Texas Medical Association . . . . . . . . . . . . . . . 686

Brazos Valley Medical Association . . . . . . . . . . . . . . . 689

The East Texas Medical Association . . . . . . . . . . . . . . 690

EDITORIAL—

A State Board of Health for Texas . . . . . . . . . . . . . . . 691

For "Durin' the War" . . . . . . . . . . . . . . . . . . . . . 693

A Flop Extraordinary . . . . . . . . . . . . . . . . . . . . . 695

The Texas Medical News . . . . . . . . . . . . . . . . . . . . 699

Our Birthday . . . . . . . . . . . . . . . . . . . . . . . . . 702

"A Convenient Memory," Indeed . . . . . . . . . . . . . . . . 702

MEDICAL NEWS AND MISCELLANY—

Many Interesting Paragraphs . . . . . . . . . . . . . . . . . . 703

PUBLISHERS' NOTES . . . . . . . . . . . . . . . . . . . . . . 708

